# Posterior localized nodular scleritis mimicking malignancy, a case report and clinical approach

**DOI:** 10.1093/omcr/omae146

**Published:** 2024-11-25

**Authors:** Mehrdad Motamed Shariati, Farid Shekarchian, Aliakbar Sabermoghaddam, Mitra Karimi Amir Abadi, Nasser Shoeibi

**Affiliations:** Eye Research Center, Mashhad University of Medical Sciences, Khatam Al-Anbia Eye Hospital, Gharani Boulevard, Mashhad, Iran; Eye Research Center, Mashhad University of Medical Sciences, Khatam Al-Anbia Eye Hospital, Gharani Boulevard, Mashhad, Iran; Eye Research Center, Mashhad University of Medical Sciences, Khatam Al-Anbia Eye Hospital, Gharani Boulevard, Mashhad, Iran; Eye Research Center, Mashhad University of Medical Sciences, Khatam Al-Anbia Eye Hospital, Gharani Boulevard, Mashhad, Iran; Eye Research Center, Mashhad University of Medical Sciences, Khatam Al-Anbia Eye Hospital, Gharani Boulevard, Mashhad, Iran

**Keywords:** posterior scleritis, choroidal tumors, multimodal imaging, posterior uveitis

## Abstract

**Aim**: To report a patient with choroidal bulging, sub-retinal fluid, and optic nerve head (ONH) swelling who was finally diagnosed with focal nodular posterior scleritis. **Case report**: A 51-year-old male patient presented to us with acute painful visual loss of his left eye (LE) from 3 days ago. The best-corrected distance visual acuity (BCDVA) was 20/20 and hand motion (HM) for the right eye (RE) and LE, respectively. Fundus examination of the LE showed ONH swelling, choroidal bulging, multiple patches of subretinal fluid accumulation, and retinal pigment epithelial (RPE) corrugations. Orbital and brain MRI showed a retrobulbar nodular mass with gadolinium enhancement at the optic nerve and sclera junction. Oncology and rheumatology work-ups were unremarkable. With the clinical diagnosis of nodular posterior scleritis oral prednisolone 50 mg/Kg was started. **Conclusion**: Posterior scleritis is an uncommon inflammatory condition that could be misdiagnosed with choroidal tumors, posterior uveitis, and orbital inflammation.

## Introduction

Scleritis is the inflammation of the sclera and episcleral tissues. According to the anatomical site of involvement relative to the equator of the eyeball, the disease is divided into anterior and posterior scleritis, which include 98% and 2% of cases, respectively [1].

Posterior scleritis is a rare vision-threatening inflammatory condition that usually presents with headache, orbital pain, and visual loss. Scleral involvement is mostly diffuse. The local nodular form of posterior scleritis is scarce [2]. These patients ‘most common funduscopic findings are optic nerve head swelling, subretinal fluid, and choroidal bulging [3]. The challenging issue in this rare category of patients is misdiagnosis with choroid tumors such as metastasis and amelanotic melanoma, as well as a wide range of differential diagnoses causing nodular scleritis [4].

In this study, we aim to report a patient with choroidal bulging, sub-retinal fluid, and optic nerve head (ONH) swelling who was finally diagnosed with focal nodular posterior scleritis. Furthermore, we will discuss the clinical approach to a patient suspected of posterior scleritis.

## Case report

**Figure 1:**
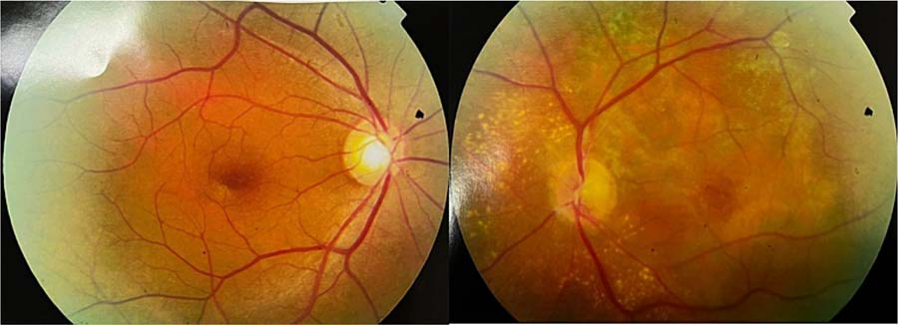
Fundus photograph of the LE showed ONH swelling, choroidal bulging, and multiple patches of subretinal fluid accumulation. Fundus photograph of the right eye revealed no abnormality.

A 51-year-old male patient presented to us with acute painful visual loss of his left eye (LE) from 3 days ago. The best-corrected distance visual acuity (BCDVA) was 20/20, and hand motion (HM) detection for the right eye (RE) and LE, respectively. The ocular movement was normal in both eyes. Anterior segment examination was unremarkable for both eyes. The LE fundus examination showed ONH swelling, choroidal bulging, multiple patches of subretinal fluid accumulation, and retinal pigment epithelial (RPE) corrugations (Fig. 1). Fundus examination of the RE was unremarkable.

**Figure 2:**
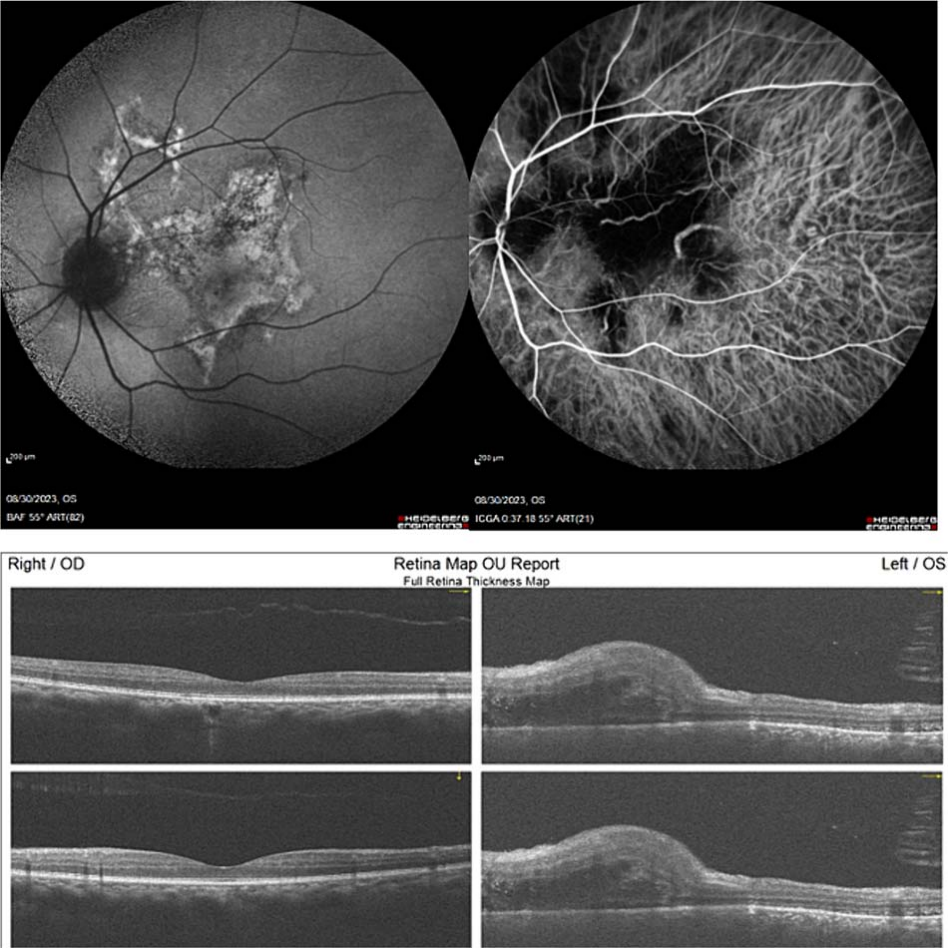
BAF (upper left) and ICGA (upper right) showed a macular abnormality with a geographic pattern. OCT images of both eyes (lower row) revealed mild RPE and choroidal bulging, RPE hyper-reflectivity with back shadowing, subretinal and intraretinal fluid accumulation in the left eye and no remarkable finding in the right eye.

**Figure 3:**
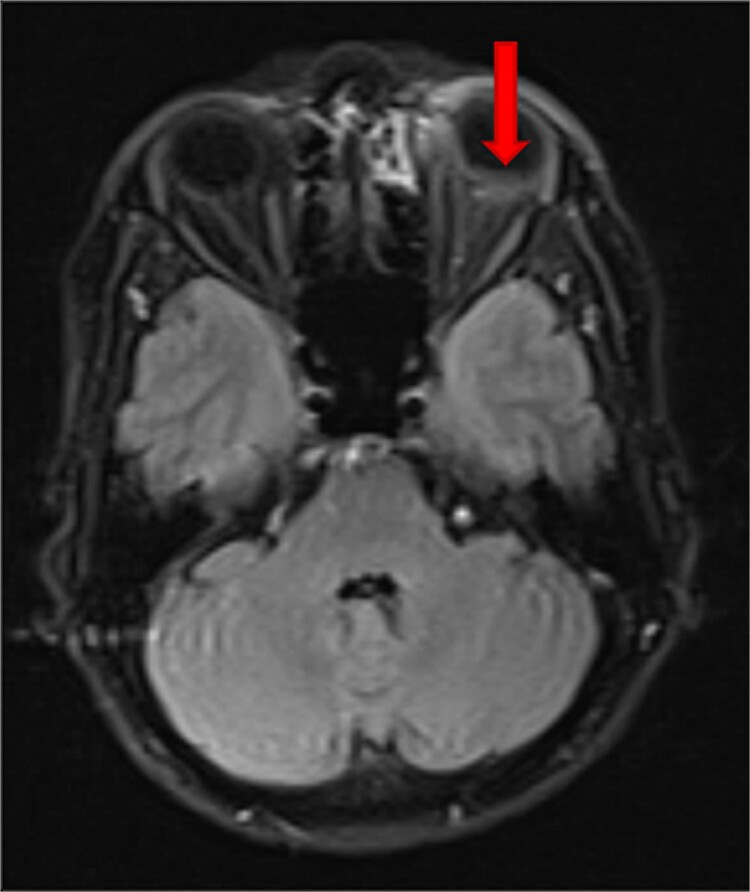
Axial brain and orbital T1-weighted fat-suppressed MRI with gadolinium enhancement showed a retrobulbar nodular mass with gadolinium enhancement at the junction of the optic nerve and sclera (arrow).

We used multimodal imaging including Optical coherence tomography (OCT) (OptoVue, Inc., Fremont, CA, USA, software version: 2018,0,0,18), fundus blue-autofluorescence (BAF), fluorescein angiography (FA) (Heidelberg Eye Explorer version 1.9.13.0, Spectralis Viewing Module 6.5.2.0; Heidelberg Engineering), Indocyanin green angiography (ICGA), and B-scan ultrasonography for further evaluation. Besides, orbital and brain MRIs with gadolinium enhancement were ordered. The OCT image revealed a mild RPE and choroidal bulging, RPE hyper-reflectivity with back shadowing, subretinal and intraretinal fluid accumulation, and mild retinal thickening. A geographic area of macular hypocyanescence was apparent in the ICGA image of the left eye. BAF showed a geographic area with a speckled autofluorescence pattern at the macula (Fig. 2). Optic nerve enlargement was found in the B-scan ultrasonography. In FA images, vascular leakage was apparent at the ONH (hot disc). Besides, a geographic patchy hypofluorescent area with speckled hyperfluorescent margins with a size of three disc diameters (DD) was detected. Orbital and brain MRI showed a retrobulbar nodular mass with gadolinium enhancement at the junction of the optic nerve and sclera (Fig. 3). An oncology consultation was done with no remarkable finding.

**Figure 4:**
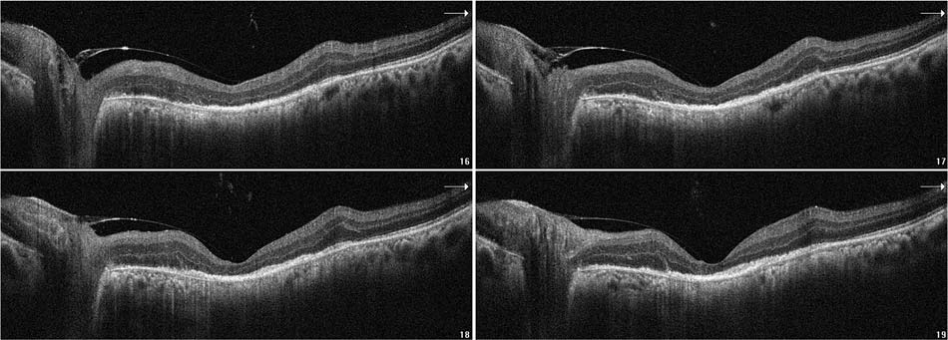
B-scan OCT images of the LE showed marked RPE and outer retinal atrophy with no SRF three weeks after the presentation.

Considering the suspicion of malignancy and the presence of an enhancing nodular mass in the orbit, the patient underwent transconjunctival lateral orbitotomy one week after the presentation. A pink localized scleral nodule with edematous tenon was found. Sub-tenon triamcinolone acetonide was injected with the clinical diagnosis of nodular posterior scleritis. The patient refused admission and intravenous corticosteroid injection as the treatment order. Oral prednisolone 50 mg/Kg was started. Rheumatology consultation and screening lab results, including PPD test (tuberculosis), chest X-ray, serum ACE level (sarcoidosis), and C-ANCA level (Wegner granulomatosis), were unremarkable. At the last follow-up examination (one week after the surgery), the patient’s BCDVA was 20/20, and counting fingers at 2 meters for the RE and LE, respectively. Furthermore, SRF was absorbed, and the macula became atrophic (Fig. 4). Oral prednisolone was tapered off slowly for three months.

## Discussion

**Figure 5:**
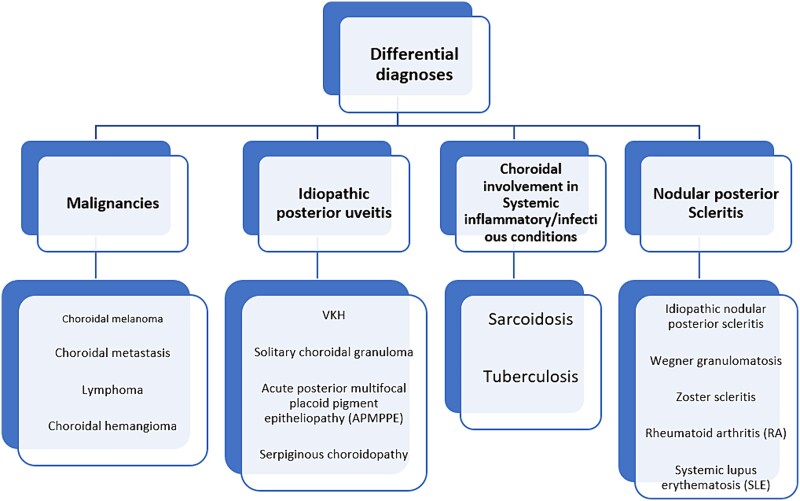
Differential diagnoses and clinical approach to a patient with unilateral choroidal bulging, subretinal fluid, and ONH swelling.

Posterior scleritis is an uncommon inflammatory condition that could be misdiagnosed with choroidal tumors, posterior uveitis, and orbital inflammation [5]. The mean age of onset is 30 to 50 years old based on the results of previous studies [1, 4]. The association of posterior scleritis with systemic inflammatory conditions has been reported in 17% to 37% [1, 6]. Regarding the site of inflammation, the disease usually manifests with painful visual loss. Common funduscopic findings include ONH swelling, subretinal fluid accumulation, choroidal congestion, and RPE undulations [7]. As we showed in Fig. 5, a wide range of differential diagnoses has to be addressed in systemic work-ups [1, 4, 7–9].

Malignancies including choroidal melanoma, metastasis, lymphoma, hemangioma, idiopathic posterior uveitis, choroidal involvement in systemic conditions such as tuberculosis and sarcoidosis, and nodular posterior scleritis are the primary differential diagnoses [1, 4, 7–9]. A complete history taking, physical examination, and collaboration with rheumatology and oncology experts seem necessary for the correct approach to these patients. Furthermore, orbital and brain MRI and multimodal posterior segment imaging are helpful. Enhanced depth imaging OCT (EDI-OCT), B-scan ultrasonography, FA, and indocyanine green angiography (ICGA) are necessary. We summarized the main clinical and imaging findings in posterior scleritis and its differential diagnosis in Table 1 [6–8, 10–12].

**Table 1:** Clinical and imaging findings in posterior scleritis and its differentials.

	**Clinical findings**	**OCT**	**B-scan ultrasound**	**Angiography**
Posterior nodular scleritis	Optic disc edema, Choroidal bulging, Choroidal fold, A variable amount of vitritis, usually unilateral	RPE undulations, Choroidal congestion, Subretinal fluid	Localized thickening of the posterior sclera, edematous tenon (T-sign), optic nerve enlargement	Early leakage with late pooling in FA
Choroidal melanoma	Choroidal mass, subretinal fluid, usually unilateral	Dome-shaped elevation of the choroid, subretinal fluid	Choroidal mass with low to medium internal reflectivity	Double-circulation pattern
VKH	Panuveitis, ONH edema, subretinal fluid, usually bilateral	Subretinal fluid with fibrinous septa, choroidal congestion	Serous retinal detachment	Delayed choroidal circulation, Disc hyper fluorescence, hypocyanescence dark dots (HDD) in ICGA
APMPPE/serpiginous	Multifocal yellowish subretinal lesions, ONH edema, usually bilateral	Disruption of the outer retinal and ellipsoid zone, subretinal fluid	—	Geographic hypo fluorescence in ICGA across all phases of the exam
Sarcoidosis/Tuberculosis	Disc swelling, pan-uveitis, choroidal granuloma, periphlebitis	Choroidal mass, macular edema	—	Retinal vasculitis, ONH granuloma

When we diagnose posterior scleritis according to clinical and imaging findings, it must be determined whether it is primary (idiopathic) or secondary. Diagnosing the etiology can help in choosing the best treatment strategy. Systemic inflammatory conditions such as Wegner granulomatosis, RA, and SLE have been found in 17%–37% of posterior scleritis cases [13]. Serology tests, including rheumatoid factor (RF), anti-nuclear antibody (ANA), c-ANCA level, and PPD test, are helpful work-ups [14].

Idiopathic nodular posterior scleritis responds well to systemic corticosteroids by improving clinical findings and visual acuity [3]. However, in more severe conditions such as Wegner granulomatosis, immunomodulating agents might be necessary.

## Key clinical message

Posterior nodular scleritis is a rare sight-threatening inflammatory condition that could masquerade the choroidal tumors, posterior uveitis, and orbital inflammation.

## Data Availability

The datasets used during the current study are available from the corresponding author upon reasonable request.

## References

[1] Dong Z-Z , Gan Y-F, Zhang Y-N. et al. The clinical features of posterior scleritis with serous retinal detachment: a retrospective clinical analysis. Int J Ophthalmol 2019;12:1151. 31341807 10.18240/ijo.2019.07.16 PMC6629818

[2] Alsarhani WK , Abu El-Asrar AM. Multimodal imaging of nodular posterior Scleritis: case report and review of the literature. Middle East Afr J Ophthalmol 2020;27:134–8. 32874049 10.4103/meajo.MEAJO_115_20 PMC7442082

[3] Ando Y , Keino H, Nakayama M. et al. Clinical features, treatment, and visual outcomes of Japanese patients with posterior scleritis. Ocul Immunol Inflamm 2020;28:209–16. 30806525 10.1080/09273948.2019.1574838

[4] Vermeirsch S , Testi I, Pavesio C. Choroidal involvement in non-infectious posterior scleritis. J Ophthalmic Inflamm Infect 2021;11:1–8. 34705127 10.1186/s12348-021-00269-9 PMC8554953

[5] Awh C , Reichstein DA, Thomas AS. A case of giant cell arteritis presenting with nodular posterior scleritis mimicking a choroidal mass. Am J Ophthalmol Case Rep 2020;17:100583. 32095658 10.1016/j.ajoc.2019.100583 PMC7033388

[6] Taki W , Keino H, Watanabe T. et al. Enhanced depth imaging optical coherence tomography of the choroid in recurrent unilateral posterior scleritis. Graefes Arch Clin Exp Ophthalmol 2013;251:1003–4. 22395202 10.1007/s00417-012-1972-1

[7] Rossiter-Thornton M , Rossiter-Thornton L, Ghabrial R. et al. Posterior scleritis mimicking orbital cellulitis. Med J Aust 2010;193:305–6. 20819053 10.5694/j.1326-5377.2010.tb03915.x

[8] Miranda AF , Cardoso J, Marques N. et al. Isolated posterior scleritis associated with tuberculosis. Arq Bras Oftalmol 2016;79:111–2. 27224075 10.5935/0004-2749.20160032

[9] Uchihori H , Nakai K, Ikuno Y. et al. Choroidal observations in posterior scleritis using high-penetration optical coherence tomography. Int Ophthalmol 2014;34:937–43. 24398712 10.1007/s10792-013-9894-4

[10] Silpa-Archa S , Silpa-Archa N, Preble JM. et al. Vogt–Koyanagi–Harada syndrome: perspectives for immunogenetics, multimodal imaging, and therapeutic options. Autoimmun Rev 2016;15:809–19 10.1016/j.autrev.2016.04.001. 27060382

[11] Testi I , Vermeirsch S, Pavesio C. Acute posterior multifocal placoid pigment epitheliopathy (APMPPE). J Ophthalmic Inflamm Infect 2021;11:1–8. 34524577 10.1186/s12348-021-00263-1 PMC8443720

[12] Mahendradas P , Maruyama K, Mizuuchi K. et al. Multimodal imaging in ocular sarcoidosis. Ocul Immunol Inflamm 2020;28:1205–11. 32396030 10.1080/09273948.2020.1751210

[13] Smith JR , Mackensen F, Rosenbaum JT. Therapy insight: scleritis and its relationship to systemic autoimmune disease. Nat Clin Pract Rheumatol 2007;3:219–26. 17396107 10.1038/ncprheum0454

[14] Akpek EK , Thorne JE, Qazi FA. et al. Evaluation of patients with scleritis for systemic disease. Ophthalmology 2004;111:501–6. 15019326 10.1016/j.ophtha.2003.06.006

